# Postural control exercise without using the upper limbs improves activities of daily living in patients with stroke

**DOI:** 10.3389/fresc.2023.1124515

**Published:** 2023-04-11

**Authors:** Akio Kamijo, Chisato Furihata, Yuki Kimura, Isamu Furuhata, Takeshi Ohtani, Takeshi Miyajima

**Affiliations:** ^1^ Nagano College of Nursing, Division of Basic & Clinical Medicine, Komagane, Japan; ^2^ Azumino Red Closs Hospital, Division of Rehabilitation, Azumino, Japan; ^3^ Matsumoto Nakagawa Hospital, Division of Rehabilitation, Matsumoto, Japan

**Keywords:** stroke, rehabilitation, intervention, recovery of function, postural control, activity of daily living (ADL)

## Abstract

**Introduction:**

Stroke is one of the most common neurological disorders worldwide. Stroke survivors have restricted activities of daily living (ADL) and lower functional independence measures (FIM) after disease onset. Recovery of postural control abilities in patients with stroke is one of the most important therapeutic goals. In this study, we examined the differences in the FIM motor items between groups that performed postural control exercises with the upper limb and those that performed postural control exercises without the upper limb.

**Methods:**

The medical records of patients with stroke admitted and discharged from the Recovery Rehabilitation Unit at Azumino Red Cross Hospital between 2016 and 2018 were reviewed. We retrospectively investigated the relationships between postural control exercises with or without upper limbs, FIM motor items at admission and discharge, and percentage of gait acquisition at discharge.

**Results and Discussion:**

Among the thirteen FIM motor items, nine (bathing, dressing the upper body, dressing the lower body, toileting, transfers [bed, chair, and wheelchair], transfers [toilet], transfers [tub or shower], locomotion, and climbing of stairs) were significantly different between the two groups (those who performed postural control exercises with the upper limb and those who performed postural control exercises without the upper limb). Patients with stroke who performed postural control exercises without the upper limbs showed a higher percentage of gait acquisition. Touch contact during quiet standing reduces body sway and the associated fluctuations. However, continual practice of postural control with a small degree of body sway for a long period after a stroke would result in decreased pressure on the sole. This may hinder postural control relearning. Touch contact also reduces anticipatory postural adjustment, which may limit the improvement in balance ability during physical exercise. Postural control exercises without the upper limbs improve postural control ability and may be beneficial from a long-term perspective.

## 1. Introduction

Stroke is one of the most common neurological disorders worldwide. Stroke survivors have restricted activities of daily living (ADL) and lower functional independence measures (FIM) after disease onset. Rehabilitation approaches can help patients with stroke recover functionally and improve their quality of life. Recently, various neurotechnology-supported interventions have been developed, including robotics, muscle electrical stimulation, brain stimulation, and brain-computer/machine interfaces (1). These approaches are effective for upper-limb motor restoration, including hand and arm movements, and induced neuroplastic changes (2–4). In addition to improving upper extremity and gait functions, which require postural control, they are also important for regaining ADLs.

Postural control is a complex sensorimotor skill designed to support postural orientation and equilibrium (5). Postural control deficits are one of the factors that impede ADLs in patients with stroke. If postural control ability is limited, problems may occur not only in walking as a means of transportation but also in various other situations such as transferring between a wheelchair and a bed, manipulating a lower garment during toilet operations, and dressing oneself. Therefore, in rehabilitation, the recovery of postural control abilities in patients with stroke is one of the most important therapeutic goals.

The center-of-pressure (COP) fluctuations during quiet standing are known indicators of postural stability, and it has been reported that COP fluctuations and postural sway are reduced by light touch (6, 7). Even in patients with stroke, touching something to maintain a quiet standing position reduces the range of COP fluctuations more than standing alone (8). However, if only a narrow COP range is achieved by touching something in a rehabilitation setting, it is conceivable that ADLs may be limited because of poor postural control in situations where there is nothing to touch. We believe that patients should practice postural control under more difficult conditions to make their lives safer, and that improving their ability to control their posture without their upper limbs will expand and improve their ADLs and quality of life, respectively.

Therefore, in this study, we examined the differences in the FIM motor items between two groups (those that performed postural control exercises with the upper limb and those that performed postural control exercises without the upper limb).

## 2. Materials and methods

The medical records of patients with stroke admitted and discharged from the Recovery Rehabilitation Unit at Azumino Red Cross Hospital between 2016 and 2018 were reviewed. The relationships between postural control exercises with or without upper limbs, FIM motor items at admission and discharge, and the percentage of gait acquisition at discharge were studied retrospectively. Each patient's FIM were evaluated at a monthly joint conference between the physician, physical therapist, occupational therapist, speech-language-hearing therapist, and ward nurses. The patients were divided into two groups: group A, patients who used their upper limbs during postural control exercises (e.g., sitting with grasping bed rails, standing or walking with seizing parallel bars); group B, patients who did not use their upper limbs as much as possible during postural control exercises. For the FIM motor items between the two groups, the difference in scores between the period of admission and discharge was tested using the Mann–Whitney *U* test. The percentage of women and gait acquisition at discharge were tested using Fisher's exact test. Two-tailed *P* values less than 0.05 were considered statistically significant. This study was approved by the Committee on Ethics of the Azumino Red Cross Hospital (Approval No: R04-A-11).

## 3. Results

The number of patients with stroke admitted and discharged from the Recovery Rehabilitation Unit at Azumino Red Cross Hospital from 2016 to 2018 was 170 (group A, 120; group B, 50). The average age was 74.1 ± 11.3 (group A) and 71.1 ± 13.1 (group B). The percentages of women in groups A and B were 46.7% and 46.0%, respectively, and the percentages of gait acquisition at discharge were 62.5% and 80%, respectively. The baseline characteristics of groups A and B are summarized in Table 1. There were no significant differences in age and the proportion of women between the two groups; however, the percentage of gait acquisition and the average change in FIM motor items from admission to discharge were significantly different.

**Table 1:** The basal characteristics of group A and B.

	Group A (*n* = 120)	Group B (*n* = 50)	*p*-value
Age (years)	74.0 ± 11.3	71.0 ± 13.1	0.16
Women (%)	46.7% (*n* = 56)	46.0% (*n* = 23)	0.99
Total points of FIM motor items at admission	46.7 ± 1.9	39.5 ± 2.1	0.01*
Total points of FIM motor items at discharge	66.1 ± 2.1	69.3 ± 3.0	0.49
Average change in FIM motor items from admission to discharge	19.9 ± 1.3	29.8 ± 2.0	<0.01**
Gait acquisition at discharge (%)	62.5% (*n* = 75)	80% (*n* = 40)	0.03*

*p < 0.05.

**p < 0.01.

There were no significant differences in age and proportions of women between the two groups; however, the total points of FIM motor items at admission, changes in FIM at admission and discharge, and percentage of gait acquisition at discharge were significantly different.

**Figure 1:**
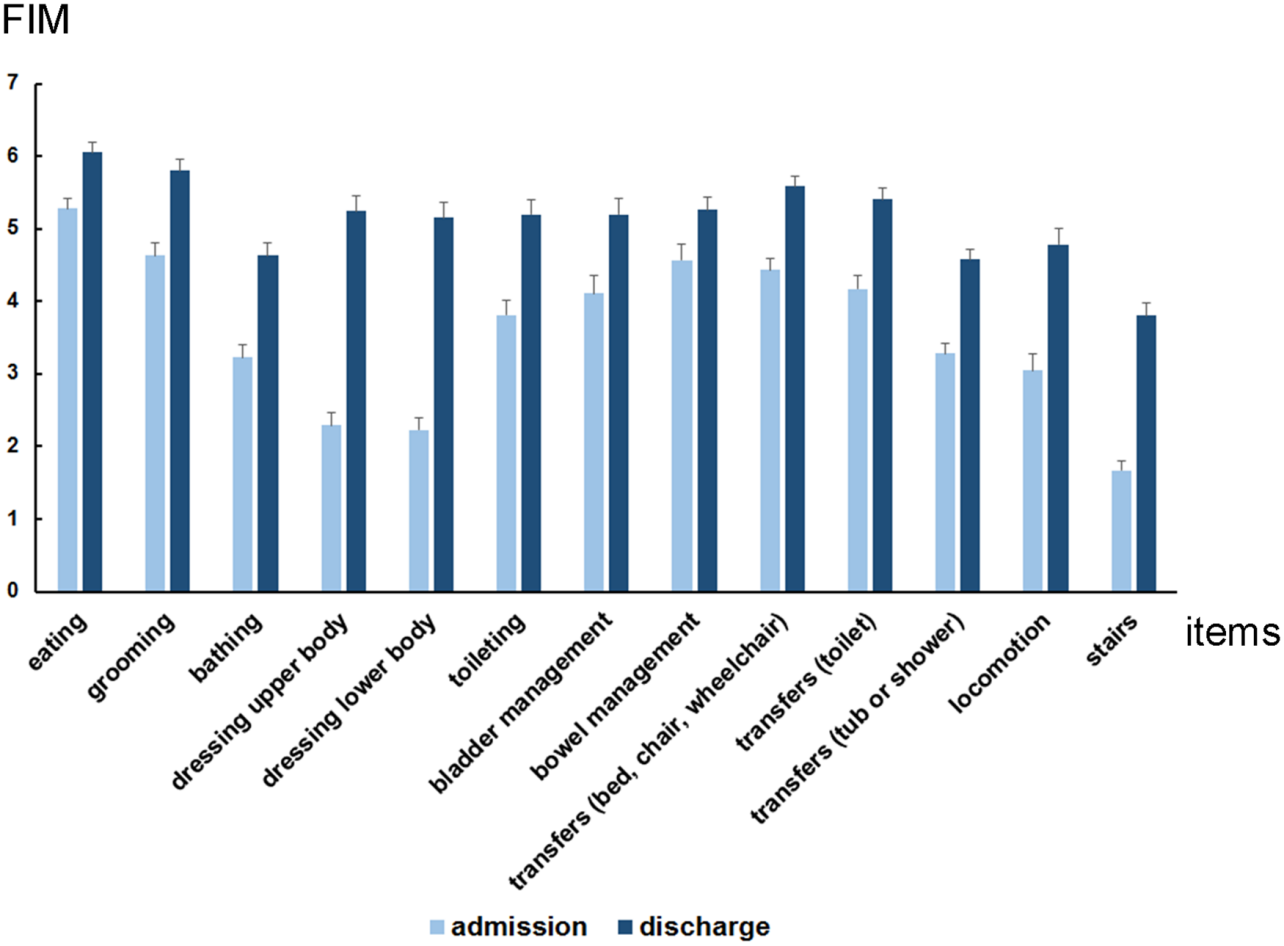
Average functional independence measures (FIM) motor items at admission and discharge in group A (patients with stroke who performed postural control exercises using the upper limbs: *n* = 120). Each FIM motor items at admission and at discharge were eating (5.3 ± 0.1 and 6.1 ± 0.1), grooming (4.6 ± 0.2 and 5.8 ± 0.2), bathing (3.2 ± 0.2 and 4.6 ± 0.2), dressing upper body (2.3 ± 0.2 and 5.2 ± 0.2), dressing lower body (2.2 ± 0.2 and 5.2 ± 0.2), toileting (3.8 ± 0.2 and 5.2 ± 0.2), bladder management (4.1 ± 0.2 and 5.2 ± 0.2), bowel management (4.6 ± 0.2 and 5.2 ± 0.2), transfers [bed, chair, wheelchair (4.4 ± 0.2 and 5.6 ± 0.1)], transfers [toilet (4.2 ± 0.2 and 5.4 ± 0.2)], transfers [tub or shower (3.3 ± 0.1 and 4.6 ± 0.1)], locomotion (3.0 ± 0.2 and 4.8 ± 0.2), stairs (1.7 ± 0.1 and 3.8 ± 0.2).

**Figure 2:**
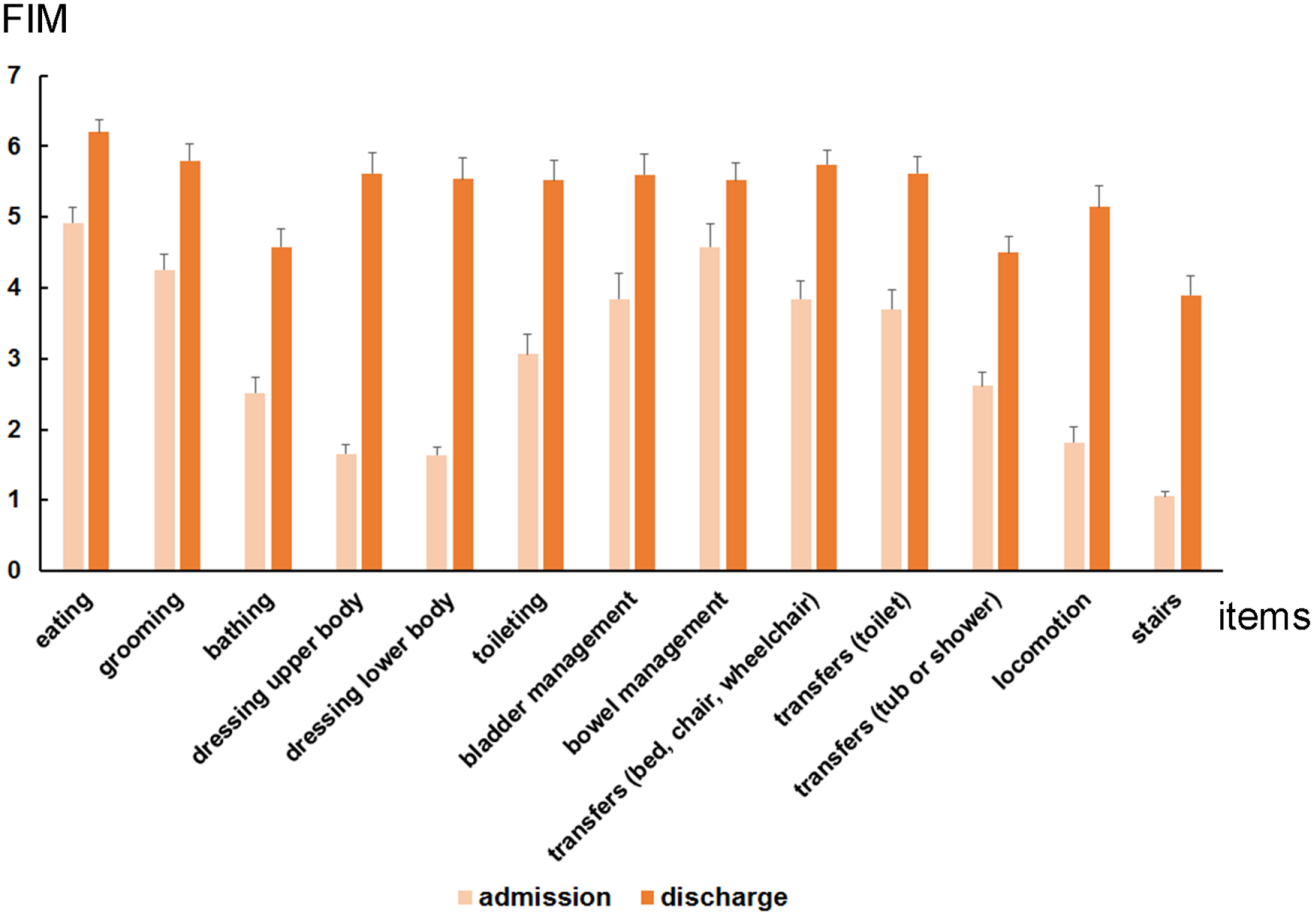
Average functional independence measures (FIM) motor items at admission and discharge in group B (patients with stroke who performed postural control exercises without using the upper limbs: *n* = 50). Each FIM motor items at admission and at discharge were eating (4.9 ± 0.2 and 6.2 ± 0.2), grooming (4.3 ± 0.2 and 5.8 ± 0.2), bathing (2.5 ± 0.2 and 4.6 ± 0.3), dressing upper body (1.7 ± 0.1 and 5.6 ± 0.3), dressing lower body (1.6 ± 0.1 and 5.5 ± 0.3), toileting (3.1 ± 0.3 and 5.5 ± 0.3), bladder management (3.8 ± 0.4 and 5.6 ± 0.3), bowel management (4.6 ± 0.3 and 5.5 ± 0.3), transfers [bed, chair, wheelchair (3.8 ± 0.3 and 5.7 ± 0.2)], transfers [toilet (3.7 ± 0.3 and 5.6 ± 0.2)], transfers [tub or shower (2.6 ± 0.2 and 4.5 ± 0.2)], locomotion (1.8 ± 0.2 and 5.1 ± 0.3), stairs (1.1 ± 0.1 and 3.9 ± 0.3).

**Figure 3:**
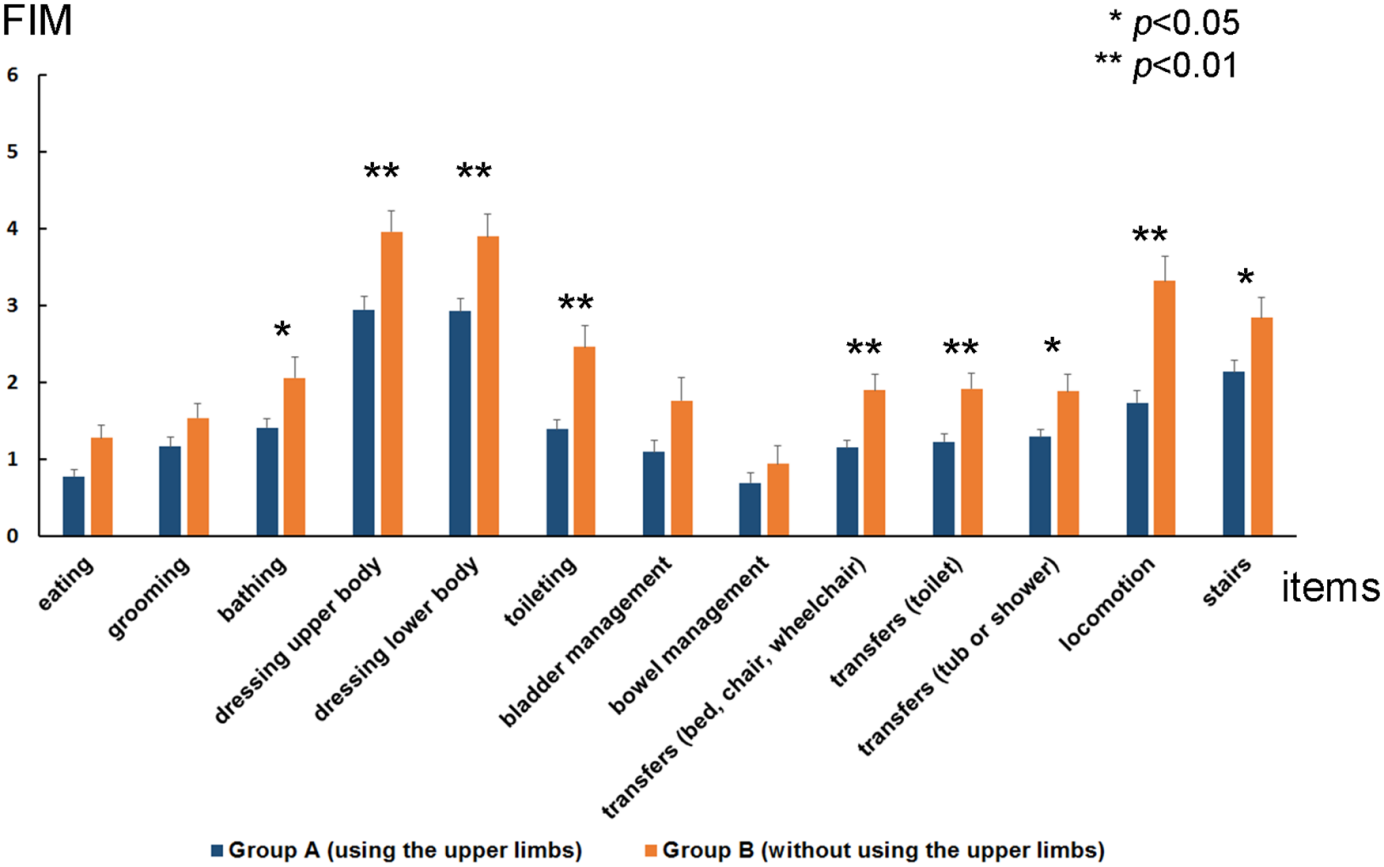
Average change in functional independence measures (FIM) motor items from admission to discharge for group A (patients with stroke who performed postural control exercises using the upper limbs: *n* = 120) and group B (patients with stroke who performed postural control exercises without using the upper limbs: *n* = 50). Each changes of FIM motor items in group A and B were eating (0.8 ± 0.1 vs. 1.3 ± 0.2), grooming (1.2 ± 0.1 vs. 1.5 ± 0.2), bathing (1.4 ± 0.1 vs. 2.1 ± 0.3), dressing upper body (3.0 ± 0.2 vs. 4.0 ± 0.3), dressing lower body (2.9 ± 0.2 vs. 3.9 ± 0.3), toileting (1.4 ± 0.1 vs. 2.5 ± 0.3), bladder management (1.1 ± 0.2 vs. 1.8 ± 0.3), bowel management (0.7 ± 0.2 vs. 1.0 ± 0.2), transfers [bed, chair, wheelchair (1.2 ± 0.1 vs. 1.9 ± 0.2)], transfers [toilet (1.2 ± 0.1 vs. 1.9 ± 0.2)], transfers [tub or shower (1.3 ± 0.1 vs. 1.9 ± 0.2)], locomotion (1.7 ± 0.2 vs. 3.3 ± 0.3), stairs (2.1 ± 0.2 vs. 2.8 ± 0.3). Among the thirteen FIM motor items, the average change in nine FIM motor items including bathing, dressing the upper body, dressing the lower body, toileting, transfers (bed, chair, and wheelchair), transfers (toilet), transfers (tub or shower), locomotion, and climbing of stairs were significantly higher in group B than in group A.

FIM motor items at admission and discharge for Group A and Group B are demonstrated in Figure 1 and Figure 2, respectively. Moreover, the FIM motor items from admission to discharge for both the groups are presented in Figure 3. In group A and B, the FIM motor items at admission were eating (5.3 ± 0.1 vs. 4.9 ± 0.2), grooming (4.6 ± 0.2 vs. 4.3 ± 0.2), bathing (3.2 ± 0.2 vs. 2.5 ± 0.2), dressing upper body (2.3 ± 0.2 vs. 1.7 ± 0.1), dressing lower body (2.2 ± 0.2 vs. 1.6 ± 0.1), toileting (3.8 ± 0.2 vs. 3.1 ± 0.3), bladder management (4.1 ± 0.2 vs. 3.8 ± 0.4), bowel management (4.6 ± 0.2 vs. 4.6 ± 0.3), transfers [bed, chair, wheelchair (4.4 ± 0.2 vs. 3.8 ± 0.3)], transfers [toilet (4.2 ± 0.2 vs. 3.7 ± 0.3)], transfers [tub or shower (3.3 ± 0.1 vs. 2.6 ± 0.2)], locomotion (3.0 ± 0.2 vs. 1.8 ± 0.2), stairs (1.7 ± 0.2 vs. 1.1 ± 0.1). Then, at discharge, there were eating (6.1 ± 0.1 vs. 6.2 ± 0.2), grooming (5.8 ± 0.2 vs. 5.8 ± 0.2), bathing (4.6 ± 0.2 vs. 4.6 ± 0.3), dressing upper body (5.2 ± 0.2 vs. 5.6 ± 0.3), dressing lower body (5.2 ± 0.2 vs. 5.5 ± 0.3), toileting (5.2 ± 0.2 vs. 5.5 ± 0.3), bladder management (5.2 ± 0.2 vs. 5.6 ± 0.3), bowel management (5.2 ± 0.2 vs. 5.5 ± 0.3), transfers [bed, chair, wheelchair (5.6 ± 0.1 vs. 5.7 ± 0.2)], transfers [toilet (5.4 ± 0.2 vs. 5.6 ± 0.2)], transfers [tub or shower (4.6 ± 0.2 vs. 4.5 ± 0.2)], locomotion (4.8 ± 0.2 vs. 5.1 ± 0.3), stairs (3.8 ± 0.2 vs. 3.9 ± 0.3).

Between the periods of admission and discharge, the average change in nine FIM motor items, which include bathing, dressing the upper body, dressing the lower body, toileting, transfers (bed, chair, wheelchair), transfers (toilet), transfers (tub or shower), locomotion, and climbing the stairs, were significantly different (*p* < 0.05) (Figure 3).

## 4. Discussion

In this study, we investigated the relationships between postural control exercises with or without the upper limbs, FIM motor items at admission and discharge, and the percentage of gait acquisition at discharge. Patients with stroke who performed postural control exercises without using their upper limbs performed better on most FIM motor items and had a higher percentage of gait acquisition. In particular, significant differences were found in the following items requiring postural control: bathing, upper body dressing, lower body dressing, toileting, transfers (bed, chair, wheelchair, toilet, tub, or shower), locomotion, and stairs, indicating that postural control practice without the upper limb improves postural balance in stroke patients in the long-term. However, there were no significant differences in the factors unrelated to the ability to control postures, such as eating, grooming, bladder management, and bowel management. Group B had lower FIM motor item scores on admission, possibly due to more severe paralysis, but showed greater improvement and a higher percentage of ambulation gain at discharge.

The decreased balance function in patients with stroke is well known, and improvements promoted by rehabilitation using various devices have been reported (9, 10). In the short term, touch contact during quiet standing reduces body sway and the associated fluctuations (11–13). However, continual practice of postural control with a small degree of COP fluctuation for a long period after stroke would result in decreased pressure on the sole, which may hinder postural control relearning. It has also been reported that touching something with the upper limbs while standing decreases anticipatory postural adjustment (APA) (14). In this study, the APA was difficult to perform during rehabilitation in Group A, which may have limited the improvement of balance ability during physical exercise. Consequently, the degree of improvement in the FIM motor items, which require postural control, may have been affected. It was also suggested that practicing in this unstable environment may have affected gait or ambulation reacquisition. Ambulation reacquisition can greatly expand ADLs after discharge (including factors not included in the FIM, such as reduced barriers and increased opportunities for going outside), which consequently improves the quality of life. Therefore, the difference in the percentage of gait acquisition shown in this study (group A, 62.5%; group B, 80%) may improve patients' post-discharge lives beyond the FIM figures. The improvement in postural control ability may also have broader effects on patients.

In the present study, we did not examine the patients' walking speed, posture, brain damage, or postural muscle electromyographic activity. Postural control involves visual, somatosensory, and vestibular function (5). However, we did not examine which of these factors improved. Furthermore, increased spatial and temporal gait variability in patients with stroke has been reported (15). Evaluation from this perspective, in addition to the percentage of gait acquisition, may lead to more accurate decisions. It has been reported that paralysis severity and gait speed are indices of fall risk (9, 16, 17), and these parameters should be considered in future studies.

It is desirable to prevent falls and ensure safety during rehabilitation; however, it is also desirable for patients to live more safely after discharge. Postural control exercises without using the upper limb may be beneficial from a long-term perspective.

## Data Availability

The raw data supporting the conclusions of this article will be made available by the authors, without undue reservation.
